# Additive Manufacturing of Information Carriers Based on Shape Memory Polyester Urethane

**DOI:** 10.3390/polym11061005

**Published:** 2019-06-05

**Authors:** Dilip Chalissery, Thorsten Pretsch, Sarah Staub, Heiko Andrä

**Affiliations:** 1 Fraunhofer Institute for Applied Polymer Research IAP, Geiselbergstr. 69, 14476 Potsdam, Germany; dilip.chalissery@iap.fraunhofer.de; 2 Fraunhofer Institute for Industrial Mathematics ITWM, Fraunhofer-Platz 1, 67663 Kaiserslautern, Germany; sarah.staub@itwm.fraunhofer.de (S.S.); heiko.andrae@itwm.fraunhofer.de (H.A.)

**Keywords:** additive manufacturing, 3D printing, shape memory polymer, fused filament fabrication, QR code carrier, thermoplastic polyurethane, filigree structures

## Abstract

Shape memory polymers (SMPs) are stimuli-responsive materials, which are able to retain an imposed, temporary shape and recover the initial, permanent shape through an external stimulus like heat. In this work, a novel manufacturing method is introduced for thermoresponsive quick response (QR) code carriers, which originally were developed as anticounterfeiting technology. Motivated by the fact that earlier manufacturing processes were sometimes too time-consuming for production, filaments of a polyester urethane (PEU) with and without dye were extruded and processed into QR code carriers using fused filament fabrication (FFF). Once programmed, the distinct shape memory properties enabled a heating-initiated switching from non-decodable to machine-readable QR codes. The results demonstrate that FFF constitutes a promising additive manufacturing technology to create complex, filigree structures with adjustable horizontal and vertical print resolution and, thus, an excellent basis to realize further technically demanding application concepts for shape memory polymers.

## 1. Introduction

Additive manufacturing (AM) alias three-dimensional (3D) printing is increasingly gaining importance, especially because of the rapid availability and the infinite design variety of print objects. Within the commercially established AM technologies, fused filament fabrication (FFF), which is a hot-melt extrusion-based 3D printing process, is widely used [1,2,3]. It requires a virtual 3D model and appropriate slicing software to convert the model into thin layers and gain the essential printing instructions. After melting in the extruder nozzle, the polymer strand is deposited layer-by-layer on the building platform of a 3D printer by moving the nozzle along a pre-calculated path. Once deposited, the polymer hardens immediately in the desired arrangement of polymer strands, which set the final shape of an object. Since FFF is an extrusion-based technique, it easily gives access to new thermoplastic materials provided they can be processed with filaments that meet the requirements of a 3D printer. To date, many thermoplastic materials have been investigated via FFF, at which special attention was devoted to polylactic acid (PLA), acrylonitrile-butadiene-styrene copolymers (ABS), polycarbonates, and polyamides [3]. Other indispensable polymer-based AM methods include stereolithography (SLA), multi-jet fusion (MJF), selective laser sintering (SLS), and big area additive manufacturing (BAAM).

Basically, the prospect of developing new applications for 3D printing improves as new functional materials are developed [4,5]. In general, many shape memory polymers (SMPs) are thermoresponsive thermoplasts. They are able to retain an imposed, temporary shape after programming and to recover the initial, permanent shape upon exposure to an external stimulus like heat [6,7,8,9,10,11]. Today, thermoplastic polyurethanes belong to the most intensively studied shape memory polymers [12,13,14,15,16,17,18,19,20,21,22,23,24,25]. Intriguingly, there are only a few publications in which FFF has been employed as a printing method for thermoresponsive polyurethane-based SMPs, the majority of them concentrating on polyether urethanes. Obviously good printing results could be achieved by Hendrikson et al. [26]. who demonstrated that scaffolds can be produced via FFF from the polyether urethane DiAPLEX^®^ MM 3520 from SMP Technologies Inc. The scaffolds were characterized by a fiber spacing of 982 ± 11 μm, a fiber diameter of 171 ± 5 μm, and a layer height of 154 ± 2 μm. In another work, Raasch et al. reported on the extrusion of the thermoplastic polyether urethane DiAPLEX^®^ MM 4520 from the same company and used the obtained filaments to manufacture specimens out of them; the 3D objects were later examined by three-point bend tests to study the influence of annealing upon shape memory behavior [27]. In a work by Yang et al., the same material was extruded and FFF used to print parts with shape memory properties [28]. Villacres et al. fabricated tensile bars of DiAPLEX^®^ MM 4520 and proved how to influence the mechanical properties by varying geometrical parameters like print orientation and infill percentage [29]. The apparently only work on extrusion-based AM of polyester urethane so far has been reported by Monzón et al. [30], who employed a custom-made 3D printer to produce parts of Desmopan^®^ DP 2795A SMP from Covestro Deutschland AG. The setting of the individual layer height was selected to be 400 µm; the stress recovery behavior of programmed parts was studied and there was a potential seen to be used as mechanical actuators. 

Until today, plenty of applications have been suggested for SMPs [31,32,33,34,35,36,37,38,39,40]. One of these applications is switchable information carriers [41,42,43]. According to the underlying concept of SMP Tagnologies^TM^, e.g., a quick response (QR) code, which can be considered as an example for a complex two-dimensional structure, is contained in the surface of an SMP. After fabrication, the code can be converted from machine-readable to unreadable by programming. Upon thermally triggering the shape memory effect, the QR code returns into the machine-readable state. The special material behavior of information release on demand can be helpful to verify and identify counterfeit products [41] or to supervise cold chains [44]. In the past, the preparation of information carriers turned out to be labor-intensive, since several manufacturing steps had to be passed through. In fact, once an SMP was processed via, e.g., injection molding, “guest diffusion” had to be applied to achieve a surface-specific coloration and laser treatment to generate a two-dimensional code in the polymer surface, before the information carrier could be obtained in its final shape by going through a cutting process [41]. Alternatively, an SMP can be coated and a code engraved into the resulting top layer, followed, e.g., by laser cutting [45,46]. Since the procedures are partly very complex, the primary goal of this work includes the introduction of an easier approach to fabricate QR code carriers. To keep it as simple as possible, the same polyester urethane (PEU), which was employed as base material in earlier generations of information carriers, was used. On the way to the production of QR code carriers, the individual steps of filament manufacturing and processing via FFF were examined, before appropriate programming paths were explored and the functionality of the QR code carriers was evaluated. Finally, the results of 3D printing were considered against the background of other technologies used in additive manufacturing.

## 2. Experimental Section

### 2.1. Material

The polyester urethane (PEU) Desmopan^®^ DP 2795A SMP from Covestro Deutschland AG (Leverkusen, Germany) was chosen as model compound and used as received in the form of a granulate. The hard segment of the PEU is composed of 4,4’-methylenediphenly diisocyanate and a 1,4-butanediol chain extender. The soft segment is based on poly(1,4-butylene adipate) (PBA). Further information regarding the thermal and mechanical properties of the PEU is given in previous publications [25,46,47].

### 2.2. Extrusion

**Figure 1:**
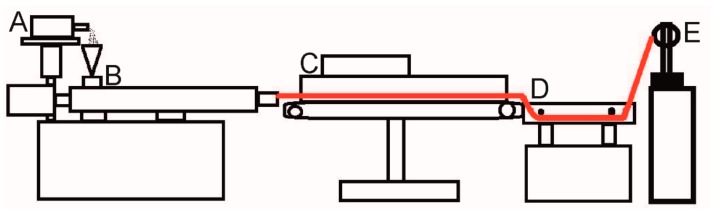
Technical drawing of an extrusion line as used for the production of PEU filaments: Material feeding system (**A**), twin screw extruder (**B**), conveyor belt (**C**), water bath (**D**), and filament winding machine (**E**). The extrudate is drawn in red.

The PEU granulate was dried at 110 °C in a Binder vacuum drying chamber VDL 53 from Binder GmbH (Tuttlingen, Germany) in order to remove water and avoid bubble formation when extruding filaments at a later stage. The thermal pre-treatment was finalized after 150 min. Subsequently, the pellets were fed into an extrusion line to produce filaments (Figure 1). 

The individual units of the extrusion line were put together in such a way that it included a volumetric material feeding system Color-exact 1000 from Plastic Recycling Machinery (Zhangjiagang City, China), a Leistritz twin screw extruder MICRO 18 GL from Leistritz AG (Nürnberg, Germany), characterized by seven heating zones and a screw length of 600 mm, a conveyor belt, a water bath and a filament winder from Brabender GmbH and Co. KG (Duisburg, Germany). The temperature of the individual heating zones of the extruder was 180, 185, 190, 195, 200, 190, and 190 °C. The screw speed of the extruder was set to 77 rpm. Initially, the PEU granulate was processed without additives. In another experiment 0.5 wt % of Irgazin^®^ Red DPP BO from Kremer Pigmente GmbH and Co. KG (Aichstetten, Germany) were added to obtain a red filament. To evaluate the quality of the filaments, the evolution of filament diameter was manually detected at regular intervals using a Vernier caliper from Fowler High Precision (Auburndale, FL, USA).

### 2.3. Virtual Design

**Figure 2:**
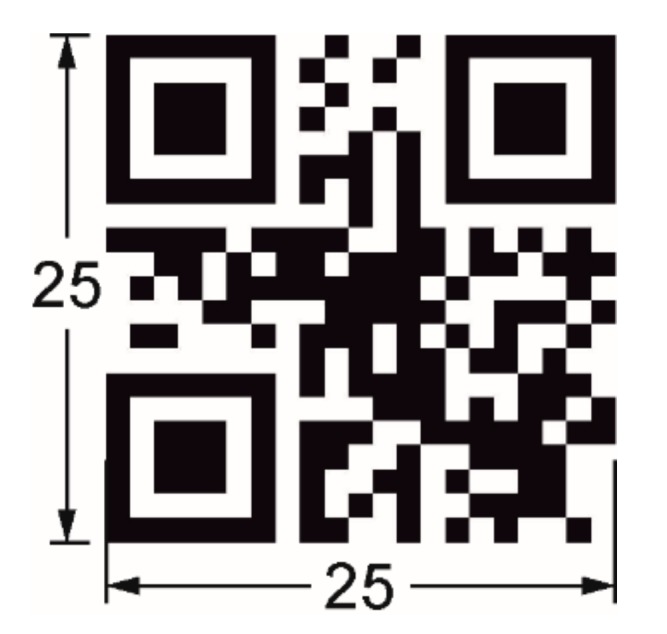
Technical drawing of a QR code, which was used as structural motif for the production of information carriers. All data are provided in mm.

The bar code generator goQR.me [48] was used to create a QR code (Reed-Solomon error correction, error correction level H) with the encoded information “Fraunhofer IAP” (Figure 2) [49].

**Figure 3:**
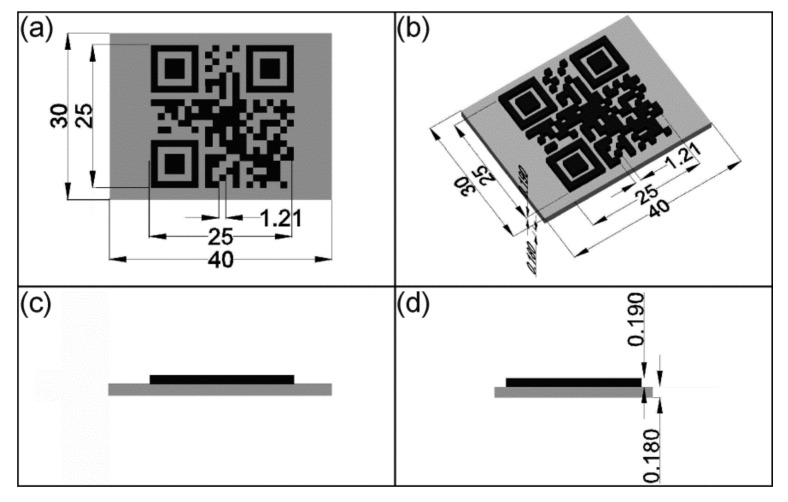
Technical drawing of a virtual QR code carrier including a substrate layer (gray color) and a structural QR code elevation (black color): Top view (**a**), isometric view (**b**), front view (**c**), and left view (**d**). All data are provided in mm.

The code was saved in the .jpeg image format and used as starting point to build a virtual information carrier by means of the vector-oriented drawing program AutoCAD from Autodesk, Inc. (San Rafael, CA, USA) (Figure 3) [50].

The edge length of the QR code was set to 25 mm (Figure 2 and Figure 3a). For the substrate layer a dimensioning of 30 mm × 40 mm was selected. As can be seen in Figure 3b–d, the QR code carrier was built up by two structural units including a substrate layer with a height of 180 µm and a structural QR code elevation with a height of 190 µm. The design of the QR code carrier as provided by Figure 3 was used for most of the experiments, which are described in this contribution. The only exception was an approach in which the target height of the substrate was reduced to 15 µm. For convenience, a terminology for the different types of QR code carriers and the associated print settings is introduced in Section 2.4. After finalizing the design, the 3D models were imported into the slicer program Cura 3.3.1 from Ultimaker B. V. (Watermolen, The Netherlands) [51]. As a result, numerically controlled codes, also denoted as G-codes, were generated, containing the instructions for the 3D printer in the form of printing paths, information with reference to the amount of extruded material and the spatially resolved printing parameters. Finally, the codes were transferred to the 3D printer. 

### 2.4. Fused Filament Fabrication (3D Printing)

Fused filament fabrication was used to produce QR code carriers with differing technical specifications. The experiments were carried out with the commercially available 3D printer Ultimaker 3 from Ultimaker B. V. (Geldermalsen, The Netherlands). The manufacturer provides an XYZ resolution for Ultimaker 3 of 12.5, 12.5, and 2.5 µm [52], defining the smallest movement that the 3D printer can make with regard to the XY plane and in the Z direction. To calibrate the print bed, the Ultimaker build plate manual leveling calibration method was carried out before the beginning of each experiment. Therefore, a calibration card characterized by a thickness of about 170 µm was used. The process included a rough leveling of the build plate followed by a fine leveling. The fine leveling was achieved with the calibration card, at which the knurled nut was adjusted at the rear center, front left, and front right of the build plate until slight friction occurred, when sliding the card between built plate and print head.

Basically, the same design of QR code carriers was used as introduced in Section 2.3. The two print heads of the FFF-printer were either equipped with two nozzles having different diameters of 100 and 400 µm, respectively, or the same diameter of 400 µm. For simplicity, the following terminology is introduced, pointing out the most relevant variations when producing QR code carriers:

- Type 1: The substrate was printed with non-dyed PEU using a 400 µm nozzle and a target layer thickness of 180 µm. The elevation was built from red PEU with a 100 µm nozzle.
- Type 2: In analogy to the type 1 QR code carrier, the substrate was printed with non-dyed PEU using a 400 µm nozzle. Again, a target layer thickness of 180 µm was selected for the substrate, but the elevation was built with red PEU employing a 400 µm nozzle.
- Type 3: Similar as in the previous cases, the substrate was printed with non-dyed PEU using a 400 µm nozzle, but this time a reduced target layer thickness of 15 µm was selected. The elevation was built with red PEU using a 400 µm nozzle.

The most relevant settings for the 3D printing processes are listed in Table 1.

### 2.5. Characterization of Thermal Properties

Dynamic mechanical analysis (DMA) was used to investigate the thermomechanical properties of the PEU. The experiments were carried out on two samples including a cylindrical granulate grain, having a diameter of 1.8 mm and a length of 4.86 mm, and a sample of a 3D printed substrate of a type 2 QR code carrier having the size of 5.15 mm × 3.8 mm × 0.19 mm. The measurements were conducted with a Q800 DMA from TA Instruments (New Castle, DE, USA) at a frequency of 10 Hz. At first, the sample was heated to 100 °C, before it was cooled to −100 °C to finalize the first heating-cooling cycle. Adjacently, the measurement cycle was repeated once more. For all experiments, heating and cooling rates of 3 °C·min^−1^ were selected and the holding time at the highest and lowest temperature was set to 10 min. The storage modulus (*E*´), loss factor (tan δ) and the glass transition temperature (*T*_g_) were determined for the second heating.

The phase transition behavior of the PEU was also studied by differential scanning calorimetry (DSC) using a Q100 DSC from TA Instruments (New Castle, DE, USA). The measurements were performed on a granulate grain, a piece of the filament and a sample of the 3D printed substrate of a type 2 QR code carrier. In any case, the sample weight was approximately 5 mg. In the experiments a sample was first cooled to −90 °C, before it was heated to 100 °C and cooled back to −90 °C, which finalized the measurement. Cooling and heating was carried out with a rate of 10 °C·min^−1^. The temperature holding time was 10 min at −90 and 100 °C, respectively. 

### 2.6. Characterization of Print Quality

Topography measurements were performed on QR code carriers using a FocusCam LV150 confocal microscope from Confovis GmbH (Jena, Germany), which was equipped with an objective lens of 5×/0.15 N.A. Any time, the sample was illuminated with a ring light. The data recorded by the focus variation microscope was evaluated with the software MountainsMap^®^ imaging topography 7.4 from Digital Surf (Besançon, France) [53]. The development of the surface profile with regard to a scanned cuboid including its surrounding was exemplarily determined for type 1 and type 2 QR code carriers. For a detailed analysis, a line was inserted along the mid-perpendicular through the cuboid. The cuboid was characterized with a step measurement to determine its height and width. 

Further microscopic investigations were carried out with the microscope Axio Scope.A1 from Carl Zeiss Microscopy GmbH (Jena, Germany) using the imaging software Zen 2.3 lite also from Carl Zeiss Microscopy GmbH [54]. The experiments were conducted to evaluate the resolution of the QR code in the XY-plane and to estimate the layer thickness of QR code carriers and thus the Z-parameter. In the latter case, a cut was made with a scalpel along the mid-perpendicular through the abovementioned cuboid. 

The printing results were also mathematically investigated. For this purpose, QR code carriers were scanned in a first step as gray value images with a resolution of 600 dpi and then loaded into the software tool ImageJ developed by Wayne Rasband (Bethesda, MD, USA) [55]. With assistance of this tool the images were cropped to the dimension of the original QR code and scaled to the corresponding resolution. The brightness and contrast were adjusted such that the influence of reflections and possible shadows was minimized. Then, the gray value images were binarized by the automatic binarization function in ImageJ. In a next step, the binarized images of the printing results were inverted. Thus, those areas where there was only the substrate of the QR code carrier were marked in black while the printed elevation parts were marked white. Adjacently, the inverse images of the printing results as well as the original QR code were imported into the software tool Paraview from Kitware, Inc. (Saratoga County, NY, USA) [56] and exported into the vtk format. The software Paraview allows mathematical operations on the values of images. The binary values of the printing results and the QR code were added up in each pixel. Due to the applied inversion for the printing results, three different gray values V were obtained in the summation (Equation (1)):

(1) $$V=\;\left\{\begin{array}{c} 0,\;erraneously\;not\;filled\;\left(blue\right)\\ 255,\;congruent\;\left(green\right)\;\;\;\;\;\;\;\;\;\;\;\;\;\;\;\;\\ 510,\;irregularly\;filled\;\left(red\right)\;\;\;\;\;\; \end{array}\right.$$

Based on these values, the print quality of the different prototypes was evaluated as a percentage p by means of Equation (2):

(2) $$p=\frac{\#\;\mathrm{pixels}\;\mathrm{of}\;\mathrm{certain}\;\mathrm{gray}\;\mathrm{value}}{\#\;\mathrm{pixels}}$$

### 2.7. Programming and Characterization of Shape Memory Properties

The programming of QR code carriers was carried out with an MTS Criterion universal testing machine from MTS Systems Corporation (Eden Prairie, MN, USA). The device was operated with a temperature chamber, which was controlled by a Eurotherm temperature controller unit. Two heating elements were located at the back of the chamber. Liquid nitrogen from a Dewar vessel was fed into the chamber under a pressure of 1.3 bar as an essential prerequisite for cooling. At the beginning of programming, a QR code carrier was clamped with a length of 25 mm, corresponding to the edge length of the QR code, in the pneumatic grips of the universal testing machine, the chamber was heated to 60 °C and a maximum force *F*_max_ of either 5 or 25 N was applied using a loading rate of 300 mm·min^−1^. The maximum distance between the outer sides of the QR code was immediately determined by means of a Vernier caliper from Fowler High Precision. The QR code carrier was then cooled to –15 °C, whereby the clamping distance was kept constant. After 10 min, the sample was unloaded and the chamber was heated to 23 °C.

A ZTNG-100B heating plate from Dr. Neumann Peltier-Technik GmbH (Neuried, Germany) was used to investigate the thermoresponsiveness of the programmed QR code carriers. Therefore, the temperature was gradually raised from 23 to 60 °C and images of the sample were taken in regular time intervals during shape recovery. After finalizing the experiment, the congruence of the QR code pattern with regard to the permanent and the recovered shape was determined and used to evaluate shape recoverability. In this connection, a similar approach was followed as described in Section 2.6, but this time gray value images were generated for the QR code carrier in its permanent and recovered shape. The gray value image of the permanent shape was regarded as the standard with which the recovered shape was compared. Therefore, the binarized gray value image of the recovered shape was inverted and added to the image containing the information of the permanent shape. The resulting gray values V were evaluated such that in the case of consistent pixels the areas were considered to be congruent while, for nonexistent pixels, the areas were regarded as incongruent (Equation (3)):

(3) $$V=\;\left\{\begin{array}{c} 255,\;congruent\;(green)\\ else,\;incongruent\;(red) \end{array}\right.$$

By analogy with the above procedure, the percentage was determined again according to Equation (2), but this time it was the measure of shape recoverability. 

A multiple cycle experiment was carried out with the MTS Criterion universal testing machine, which was equipped with a temperature chamber. For loading, a type 2 QR code carrier was clamped with a length of 25 mm, corresponding to the edge length of the QR code, in the pneumatic grips of the universal testing machine, heated to 60 °C, deformed with a rate of 300 mm·min^−1^ to a maximum clamping distance of 55 mm, before unloading was carried out at the same temperature with a rate of 150 mm·min^−1^. In total, 20 cycles of loading and unloading were conducted. In the 21^st^ cycle, the sample was loaded and the imposed shape was fixed by cooling to −15 °C. After unloading, the temperature was raised to 23 °C and the machine readability of the QR code was checked. The programmed QR code carrier was adjacently heated to 60 °C where, again, the readability of the QR code was investigated. To characterize the boundary between substrate and elevation, another programming was accomplished. In this 22^nd^ cycle, a cut was made with a scalpel along the mid-perpendicular through the abovementioned cuboid and investigated by means of light microscopy. The sample was finally heated to 60 °C and a microscopic investigation was carried out with the microscope Axio Scope.A1, which was equipped with an objective lens of 20× and 40× magnification. Following other programming scenarios, a QR code carrier was folded in the middle or rolled up at 60 °C, before it was cooled under load to −15 °C. Afterwards, the thermoresponsiveness was again followed on the heating plate when triggering the shape memory effect. Independent of the programming technique applied, the machine readability of QR codes was checked with a Samsung Galaxy S8 smartphone from Samsung Electronics (Seoul, South Korea), which was equipped with the software “Optical Reader” version 4.4.07 also from Samsung Electronics Co., Ltd [57].

## 3. Results and Discussion

**Figure 4:**
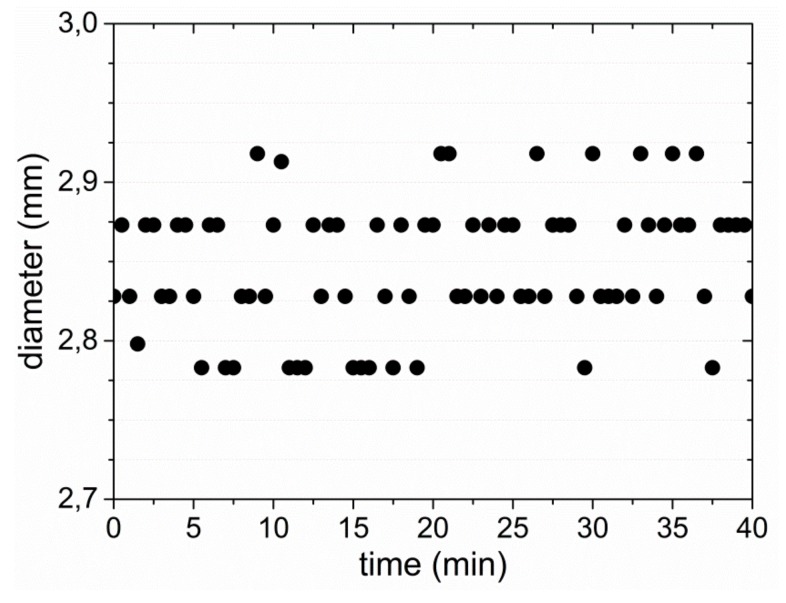
Evolution of filament diameter over time when extruding PEU. The development in measured values is also representative for an experiment in which Irgazin^®^ Red DPP BO was added during extrusion of PEU.

The melt extrusion of the physically cross-linked PEU block copolymer led to the production of a whitish filament whose color can be traced back to the presence of crystals from poly(1,4-butylene adipate) (PBA); the proof will be given below in a DSC measurement. In another experiment, 0.5 wt % of Irgazin^®^ Red DPP BO was added in the course of PEU extrusion so that a red filament could also be obtained. It is noteworthy that the two filaments had a homogenous diameter of 2.85 ± 0.07 mm, regardless of whether the dye was added or not (Figure 4).

Before starting with the 3D printing experiments, the design of the QR code carriers was developed (Figure 3). The objects were sliced to obtain the essential printing instructions. In a next step, a dual extrusion FFF process was established, in which the already obtained filaments were reprocessed to build up QR code carriers, characterized by a whitish substrate and a red QR code elevation. The most relevant settings for the 3D printing processes are provided in Table 1. 

**Figure 5:**
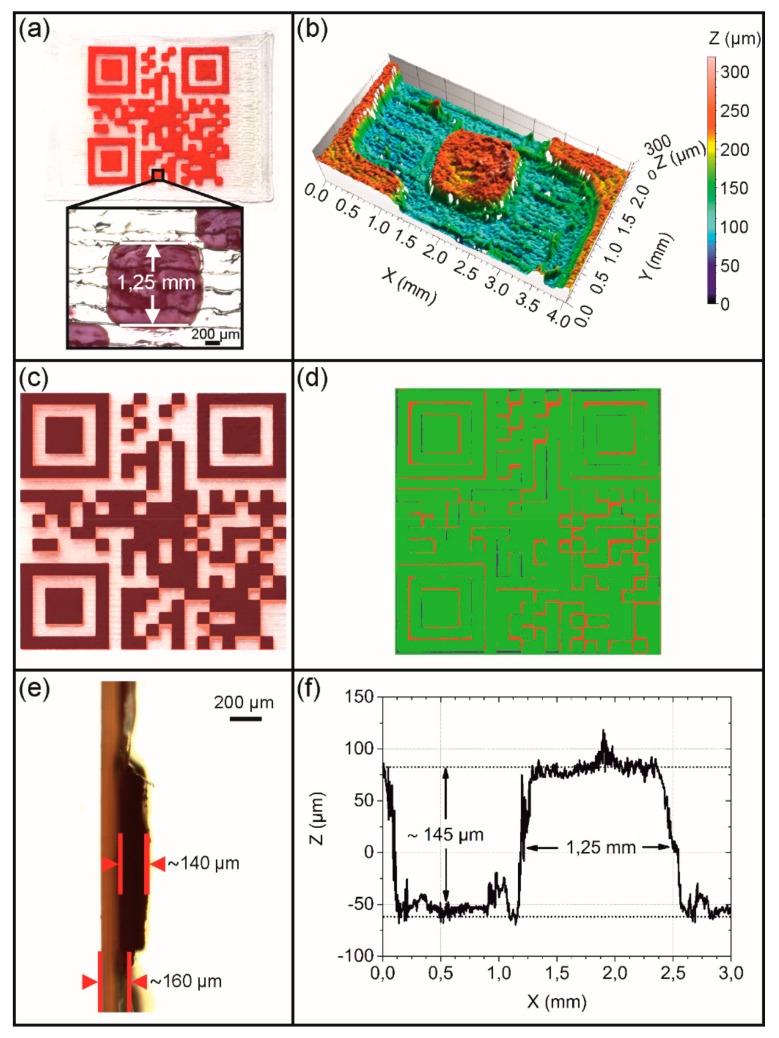
Type 1 QR code carrier as investigated by light and confocal microscopy including an evaluation of print quality: Top view and inset exhibiting a randomly selected cuboid (**a**), surface topography of the cuboid and its surrounding (**b**), superposition with a virtual QR code having a transparency of 60% (**c**), result of a mathematic calculation to determine the congruence of the virtual QR code with the physical print object: consistent print areas (green color), irregularly filled areas (red color) and unfilled print areas (blue color) (**d**), side view of a cut through the cuboid and the substrate (**e**), and the evolution of layer thickness Z with regard to the cuboid and its surrounding (**f**).

For the production of a type 1 QR code carrier, a single-layer substrate with a target height of 180 µm was printed, using the white filament and a nozzle with a diameter of 400 µm. In contrast, the QR code elevation having a virtual height of 190 µm was then built up in three layers by melting the red filament in the 100 µm nozzle and placing the resulting strands on the substrate. The printing results are portrayed in Figure 5 together with their microscopic characterization. 

**Figure 6:**
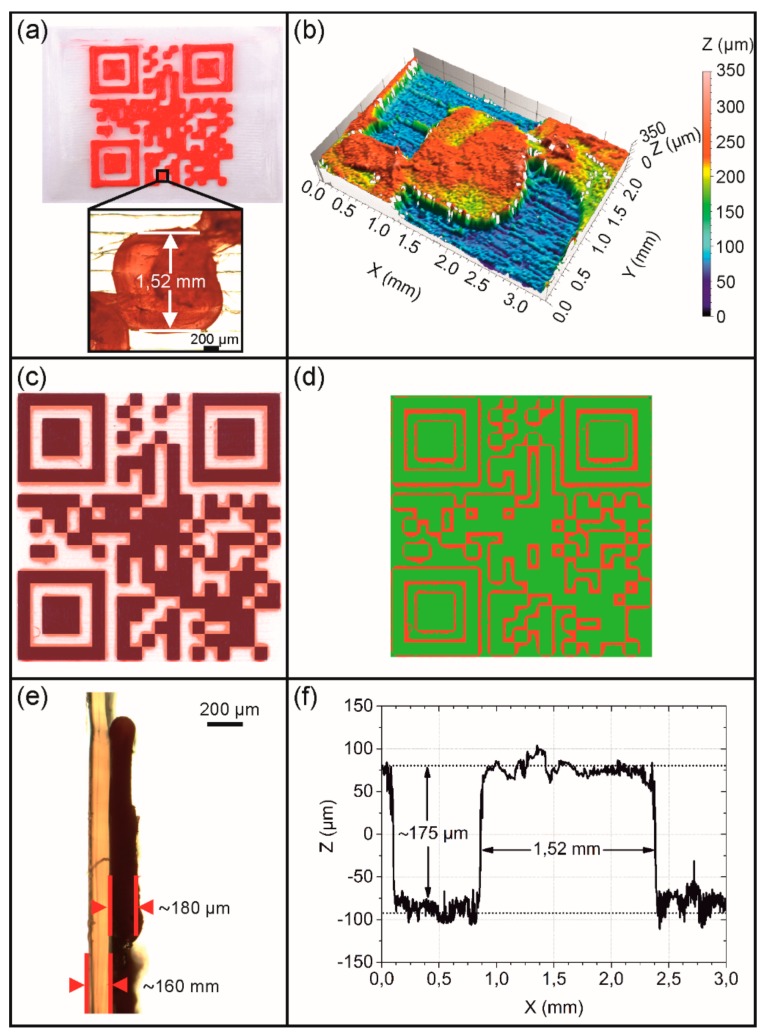
Type 2 QR code carrier as investigated by light and confocal microscopy including an evaluation of print quality: Top view and inset exhibiting a randomly selected cuboid (**a**), surface topography of the cuboid and its surrounding (**b**), superposition with a virtual QR code having a transparency of 60% (**c**), result of a mathematic calculation to determine the congruence of the virtual QR code with the physical print object: consistent print areas (green color) and irregularly filled areas (red color) (**d**), side view of a cut through the cuboid and the substrate (**e**), and the evolution of layer thickness Z with regard to the cuboid and its surrounding (**f**).

The obtained type 1 QR code carrier exhibited a good spatial resolution with respect to the XY level as exemplified by the presence of finely resolved rectangles (Figure 5a). In order to better assess the print quality with regard to the smallest structural unit of the QR code pattern, a cuboid of the finished part with a virtual edge length of 1.21 mm was microscopically examined (Figure 5a,b). Here, an edge length of approximately 1.25 mm could be determined. This value exceeded the one of our CAD model by 40 µm corresponding to 3.2% of the object dimension (Figure 3a). Basically, a deviation from the technical specification was anticipated due to slight fluctuations in filament diameter (Figure 4) and minor differences in the print bed height resulting from the calibration [58]. However, in the XY plane the print quality of the overall QR code pattern was pretty good as supported by the result of a superposition experiment, in which the virtual QR code was put with a transparency of 60% over the printing pattern (Figure 5c). In addition, a congruence measurement was carried out, subtracting the overhanging regions of the QR code elevation from the black regions of the virtual code. The result gave that 90.7% of the code areas were congruent, 8.1% were irregularly filled with red PEU and 1.2% were erroneously not filled (Figure 5d). Next, the resolution in the Z-direction was closely investigated for the same cuboid and its nearest surrounding. The substrate of the QR code carrier had a thickness of about 160 µm (Figure 5e). The averaged profile height of the elevation was determined to be approximately 145 µm, corresponding to a mean layer height of about 48 µm (Figure 5f). The layer thickness was slightly below the target value, presumably due to deficits in calibration accuracy. The production of the QR code elevation took 17 min, culminating for the whole QR code carrier in a production time of 25 min. For a faster production, the 100 µm nozzle was replaced by a 400 µm nozzle and the technical parameters were adjusted accordingly (see Table 1). As a result, a type 2 QR code carrier was obtained and examined microscopically (Figure 6).

This time, the presence of more imperfect rectangles could be witnessed in the QR code pattern (Figure 6a,b). Once more, the cuboid was studied, which was located at the same position of the QR code as in the preceding case (Figure 5a), in order to get a first impression about the precision in the XY printing plane. Here, a drastically increased edge length was determined as documented by a value of about 1.52 mm (Figure 6a), exceeding the virtual dimensions of this element by 26% (Figure 3a). The fact that the horizontal print resolution substantially deteriorated in the whole QR code area was confirmed by another superimposition experiment. As visible to the naked eye, the printed regions generously overlapped the black areas of the virtual QR code pattern (Figure 6c). Against this background, another mathematic calculation was carried out. It turned out that 77.4% of the code areas were congruent, whereas 22.6% of those code areas, in which no printing was desired, were covered with red PEU (Figure 6d). However, compared to the type 1 QR code carrier, the same substrate thickness could be verified as expected, but better control over the vertical print resolution could be achieved as indicated by an average profile height of 175 µm (Figure 6e,f). Furthermore, the production time of the QR code elevation could be drastically reduced to 3 min and 30 s so that the printing of the entire QR code carrier was finalized after 11 min and 30 s. 

Despite the abovementioned dimensional inaccuracies in the 3D printed objects, the QR codes enabled an error-free decoding with a standard smartphone, independent of which technical equipment and parameter settings were used for printing. This clearly shows that the surface contrast was sufficiently high as ensured by the processing of the differently colored filaments. 

**Figure 7:**
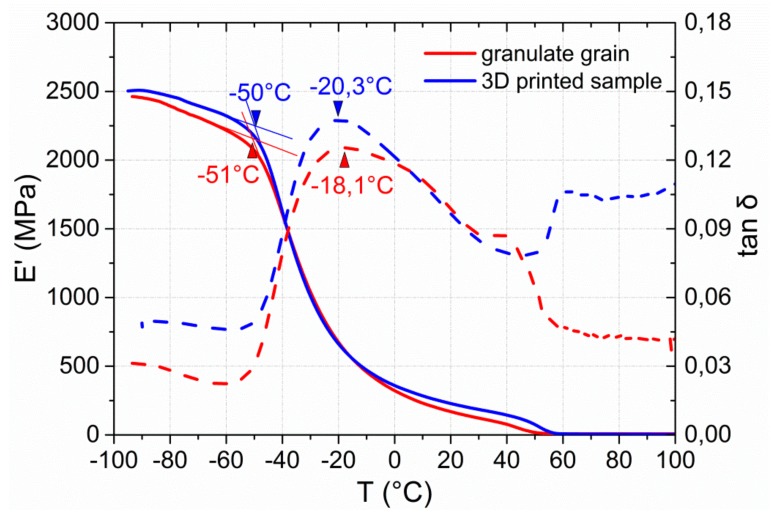
Thermal and mechanical properties of PEU as determined by DMA: Evolution of storage modulus *E*’ (solid line) and tan δ (dashed line) at the second heating of a granulate grain (red color) and the sample of the substrate of a type 2 QR code carrier as manufactured via FFF (blue color).

To investigate the influence of reprocessing via extrusion and FFF on the viscoelastic properties of the PEU, dynamic mechanical analyses were conducted. Therefore, the raw material in the form of a granulate grain was studied and compared with the thermomechanical behavior of a sample, which was taken from the 3D printed substrate of a type 2 QR code carrier. The associated temperature-dependent evolution in storage modulus E’ and in tan δ is provided by Figure 7.

**Figure 8:**
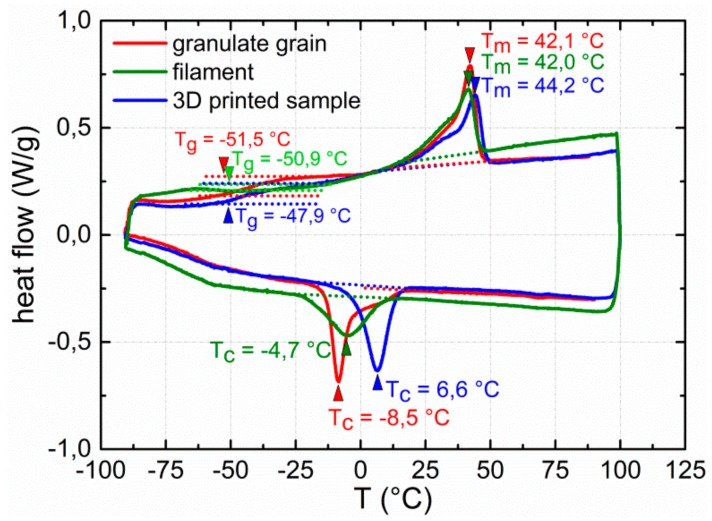
DSC thermograms of PEU: Thermal behavior of a granulate grain (red color), a piece of filament (green color) and a sample from the substrate of a type 2 QR code carrier as obtained via FFF (blue color). The thermograms are exhibited for the second heating and cooling. The individual enthalpies of melting are 25.9 J·g^−1^ (granulate grain), 21.1 J·g^−1^ (filament) and 25.1 J·g^−1^ (3D printed sample), the enthalpies of crystallization are −25.7 J·g^−1^ (granulate grain), −21.0 J·g^−1^ (filament), and −25.0 J·g^−1^ (3D printed sample).

In both cases, E’ exhibits a two-step decrease in the DMA measurement as characteristic for physically cross-linked PEU [22,59,60,61]. The investigation of the granulate grain reveals a strong drop in E’, starting at −51 °C and indicating the presence of a glass transition. The tan δ peak is located at about −18 °C. Upon further heating, a weaker decline in E’ takes place, which can be associated with the melting of PBA crystals as earlier verified for the same material [44]. The 3D printed sample shows a similarly pronounced drop in *E*’, starting again at approximately −50 °C, and a tan δ peak at −20 °C, which is in accordance with the thermal behavior of the granulate grain. In contrast, the decline in storage modulus associated with PBA melting is slightly extended toward higher temperatures. This could be related to an orientation effect as supported by reprocessing via FFF, favoring the formation of PBA crystals with higher temperature stability. In other words, the conditions under which parts of the PBA phase of PEU crystallized were expected to be more favorable for the 3D printed sample. To take another look at this, DSC measurements were carried out (Figure 8).

The DSC cooling trace of the 3D printed sample shows an exothermic signal at about 7 °C associated with the recrystallization of the PBA phase [25]. Compared with the thermal behavior of the granulate grain, the peak crystallization temperature increased by about 15 °C. This observation can be taken as further hint that strand deposition in course of 3D printing favored a better alignment of polymer chains, thus facilitating the recrystallization of PBA. In a third DSC measurement, the filament of PEU was investigated. Here, another exothermic signal associated with PBA crystallization appeared on cooling, justifying the whitish color of the filament. In this case, the peak crystallization temperature was closer to the one of the granulate grain. In turn, the DSC heating traces of the three samples show the presence of two phase transitions. The first one is located at around −50 °C and, thus, close to the point at which E’ started to drop in the DMA measurement. It is related to the glass transition temperature of the PBA phase, while the endothermic signal in between 20 and 50 °C with a maximum at around 40 °C can be assigned to the melting of PBA crystallites [25]. Here, the same trend as in the DMA measurement could be verified, but the melting peak temperature of PBA only increased by 2 °C for the 3D printed sample compared with the granulate grain. Beyond that, the melting behavior of the crystalline PBA phase appeared to be similar for the filament and the granulate grain. Most importantly, when considering both the DMA and DSC data, no further evidence was found that two-step processing, including extrusion and FFF, had a significant impact on the thermal properties of the PEU. 

**Figure 9:**
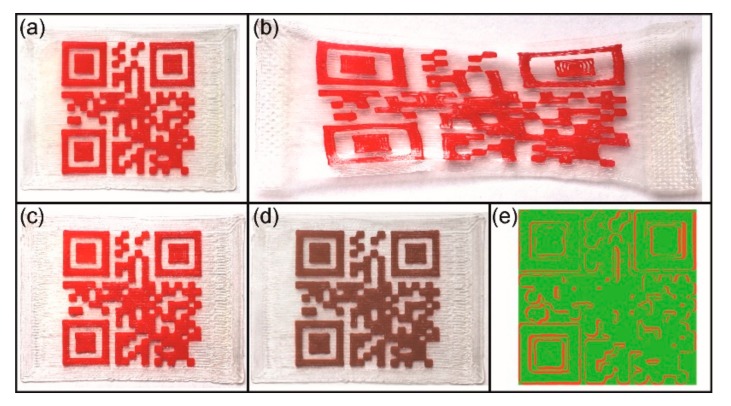
Type 2 QR code carrier: Permanent shape after 3D printing (**a**), temporary shape as obtained after programming (*F*_max_ = 5 N) (**b**), and recovered shape after heating to 60 °C (**c**). To visualize shape recoverability, the image of the permanent shape was converted to black-and-white and superimposed with a transparency of 60% on the image of the recovered shape (**d**). The result of a mathematical calculation comparing the permanent shape with the recovered shape: congruent areas (green color) and incongruent areas (red color) (**e**).

In a next step, the shape memory properties of a type 2 QR code carrier were investigated (Figure 9).

**Figure 10:**
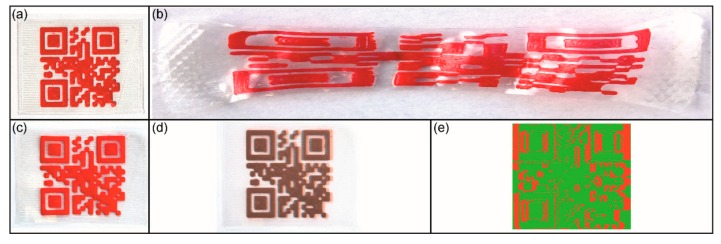
Type 2 QR code carrier: Permanent shape after 3D printing (**a**), the temporary shape as obtained after programming (*F*_max_ = 25 N) (**b**), and the recovered shape after heating to 60 °C (**c**). To visualize shape recoverability, the image of the permanent shape was converted to black-and-white and superimposed with a transparency of 60% on the image of the recovered shape (**d**). The result of a mathematical calculation comparing the permanent shape with the recovered shape: congruent areas (green color) and incongruent areas (red color) (**e**).

Therefore, the additively manufactured QR code carrier (Figure 9a) was heated to 60 °C, at which the melting of the PBA phase was completed. Subsequently, a tensile force *F*_max_ of 5 N was applied, whereupon a maximum distance length of 55 mm between the outer sides of the QR code was detected. The elongated QR code carrier was fixed by cooling below the crystallization temperature of the PBA phase and unloaded (Figure 9b). Due to changes in the design of the QR code carrier and in particular because of the much smaller structural thickness of only 160 µm, a significantly lower deformation force was required to achieve a similar QR code distortion in the programmed shape compared to an earlier generation of QR code carriers, which was characterized by a thickness of 2 mm and required a tensile force *F*_max_ of 48 N [41]. Intriguingly, the bonding was strong enough to withstand a removal of the QR code elevation from the substrate in the course of deformation. The programmed shape of the QR code carrier, which was stable at 23 °C, was characterized by the largest distance length between the outer sides of the QR code of 54 mm, speaking for the excellent shape fixity of the polymer. Due to its drastic distortion the code was no longer machine-readable. Upon triggering the shape memory effect, the QR code pattern almost completely returned to the original shape (Figure 9c), which was accompanied with the restoration of machine readability. For a more detailed study, another image analysis was carried out. Herein, the superimposed QR codes of the original shape and the recovered shape turned out to be almost identical (Figure 9d). The distinct shape recoverability was evidenced by another mathematic calculation, unveiling that 87.8% of the code areas of the permanent shape and the recovered shape were congruent (Figure 9e). Overall, the pronounced shape memory properties, which were detected in the first experimental series, raised the question if type 2 QR code carriers are able to resist even stronger deformations. To find out the answer, a similar programming experiment as described above was performed, but this time *F*_max_ was raised to 25 N (Figure 10).

As a matter of fact, the QR code carrier produced by FFF (Figure 10a) was elongated so that the outer sides of the QR code had a maximum distance of about 155 mm. After cooling below the crystallization temperature of the PBA phase and unloading, the temperature was raised to 23 °C. Here the new, even more strongly deformed shape proved to be stable (Figure 10b). The distance between the outer sides of the elongated QR code measured 153 mm in tensile direction which, again, revealed the excellent shape fixity of the polymer. It is remarkable that even in this case the QR code became machine-readable again after triggering the shape memory effect (Figure 10c), which demonstrates that the concept of information release on demand was still working. Apparently, the decoding algorithm of the smartphone was able to compensate the residual distortion. The discrepancy between the QR code pattern of the permanent shape and the recovered shape can be clearly seen in the corresponding superimposed images (Figure 10d). As quantified in one further mathematical calculation, 72.7% of the code areas were congruent (Figure 10e). Compared to the previous case, a weakening of shape recoverability was expected due to the stronger deformation applied. It can be assumed that this phenomenon of growing residuals with increasing elongation can be traced back to the flow of amorphous segments in the polymer [62].

To determine the degree of deformation, at which the QR code was no longer machine-readable, another type 2 QR code carrier was deformed at 60 °C with a rate of 0.5 mm·min^−1^ while the machine readability of the QR code was regularly checked. It turned out that the QR code became unreadable as soon as a distance length of 30 mm between the outer sides of the QR code was exceeded. 

**Figure 11:**
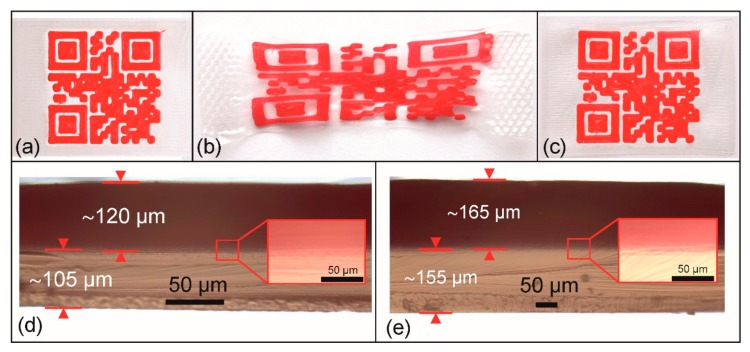
Type 2 QR code carrier: Permanent shape after 3D printing (**a**), initial clamping distance = 25 mm), temporary shape as obtained after 20 loading-unloading cycles (maximum clamping distance = 55 mm) at 60 °C, followed by programming (**b**), and the recovered shape after heating to 60 °C in the 21^st^ cycle (**c**). Microscopic investigation of a cut through the cuboid and the substrate as examined in the 22^nd^ cycle for the programmed shape (**d**) and the recovered shape (**e**); the insets show an enlarged view of the boundary between the substrate (below) and the elevation (above).

In an attempt to study the reliability of shape memory properties, a type 2 QR carrier was exposed to a multiple cycle experiment (Figure 11). 

The additively manufactured QR code carrier (Figure 11a) was loaded to a clamping distance of 55 mm and unloaded twenty times, before a loading at 60 °C and unloading at −15 °C was accomplished. In this 21^st^ cycle, the QR code was non-decodable at 23 °C and characterized by a maximum distance length of 55 mm between its outer sides (Figure 11b). Triggering the shape memory effect by reheating to 60 °C resulted in shape recovery as accompanied with the restoration of the machine-readable code, characterized by a maximum edge length of 26.8 mm (Figure 11c). This unequivocally demonstrates the reliability of the concept of switchable information carriers. In the ensuing 22^nd^ cycle, the thermomechanical treatment of the previous cycle was repeated, but neither micro cracks nor delamination could be microscopically detected at the boundary between the substrate and the elevation both for the programmed shape (Figure 11d) and for the recovered shape (Figure 11e). This finding indicates good layer coalescence. As expected, the triggering of the shape memory effect led to an increase in layer thickness. In fact, a recovery from 105 to 155 µm for the substrate and from 120 to 165 µm for the elevation could be verified. 

**Figure 12:**
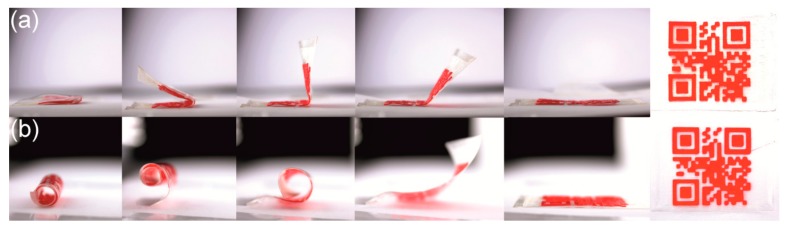
Thermoresponsiveness of type 2 QR code carriers, which were deformed in a folding approach (**a**) and a rolling approach (**b**): Programmed shapes (left), sequential shape recovery when placed on a 60 °C hot heating plate (images 2–4) and recovered shapes (images 5–6).

Next, the deformation scenarios for the programming of QR code carriers were expanded toward rolling and bending, before the respective thermoresponsivity was studied (Figure 12).

Therefore, two of our type 2 QR code carriers were heated to 60 °C, at which the PBA phase of the PEU was completely amorphous. The first sample was folded in the middle (Figure 12a), the other was rolled up (Figure 12b). The fixation of the resulting temporary shapes was then achieved on cooling below the crystallization temperature of the PBA phase. After unloading, the QR code carriers were placed on a heating plate, which had a temperature of 60 °C. In both cases it took about 10 s to finalize shape recovery, which again was accompanied with the restauration of the machine-readable QR codes, thus demonstrating that the concept is not restricted to deformation scenarios like elongation or compression [41,63].

**Figure 13:**
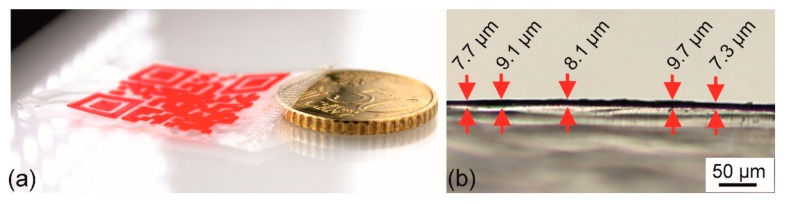
Type 3 QR code carrier: Illustration of size and thickness in comparison with a 50 euro cent coin, which is characterized by a height of 2 mm (**a**) and image of a light microscopic investigation to estimate the thickness of the QR code carrier (**b**).

Following another design approach, the dimensions of the QR code carrier were altered by drastically reducing the target substrate thickness from 180 to 15 µm. As a result, a type 3 QR code carrier was obtained (Figure 13).

The production time of the type 3 QR code carrier was about 11 min 30 s, corresponding to the processing time of the type 2 QR code carriers. To illustrate the low thickness, a 50 euro cent coin having a thickness of 2 mm was placed next to it (Figure 13a). As determined in a microscopic measurement, the thickness of the PEU substrate varied from about 7 to 10 µm (Figure 13b). 

It is also worth mentioning that the weight of all QR code carriers described herein was significantly lower compared to earlier generations of prototypes, which were obtained by other processing techniques [41,45,46]. In direct comparison with each other, the introduced type 1 and type 2 QR code carriers were approximately weighing 340 mg while the weight of the type 3 QR code carrier was 100 mg and, thus, significantly lower, qualifying it for applications, in which the costs for transport must be kept under control. 

**Figure 14:**
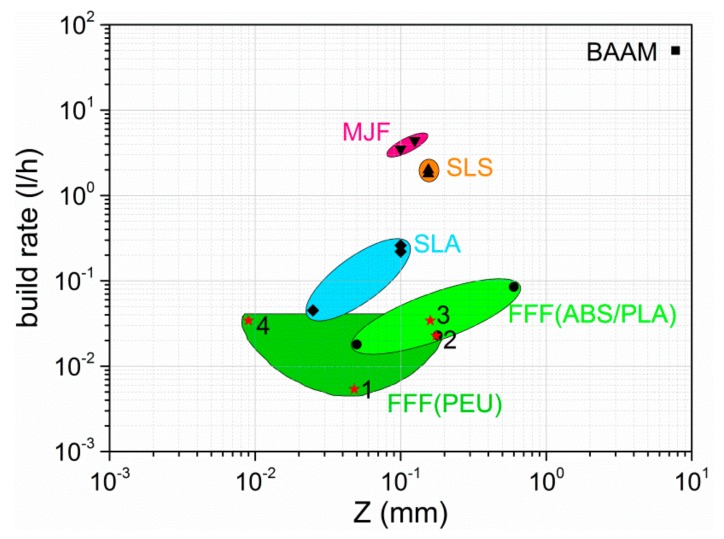
Build rate versus layer thickness for common additive manufacturing processes. The initial data was extracted from Quinlan et al [64]. The red stars represent data points for the QR code elevation as part of the type 1 QR code carrier (1) and the type 2 QR code carrier (2), while the remaining data points refer to the substrate of the type 1 and type 2 QR code carrier (3) and the type 3 QR code carrier (4).

For the purpose of comprehensive consideration, the printing results described herein were compared with those of other printing materials, which were processed by FFF, and additionally with the results of other 3D printing techniques. For convenience, the same approach was followed as by Quinlan et al. [64], who compared polymer-based processes like fused filament fabrication (FFF), stereolithography (SLA), big area additive manufacturing (BAAM), multi-jet fusion (MJF) and selective laser sintering (SLS) with particular emphasis on build rate and layer thickness, the latter of which can be considered as a measure of Z-direction accuracy. The corresponding results are supplied in Figure 14.

It can be clearly seen that SLA, BAAM, MJF, and SLS provide higher build rates compared with FFF. In turn, FFF makes it particularly possible to control the layer thickness, namely, the Z-parameter, over quite a wide range as apparent for printing materials like ABS and PLA. However, the data points introduced for the presented QR code carriers do also cover a broad area, which in parts overlaps with the already existing data for FFF. Due to the printing result of the thin layer as evident for the substrate of the type 3 QR code carrier, a data point emerges, defining the lowest value for Z. Interestingly, this reasonably good print resolution could neither be achieved by other groups, working on shape memory polyurethanes using extrusion-based AM techniques [26,27,28,29,30,65,66] nor by other researchers who utilized those 3D printing techniques, which were described by Quinlan et al. [64]. Admittedly, two-photon lithography (2PL) is another AM technology, which was not included in our considerations, but allows obtaining 3D objects, which are characterized by even smaller layer thicknesses of 0.2 to 0.3 µm [67]. Although being particularly advantageous in resolution, the good print results of 2PL are at the expense of the build rate. Therefore, a compromise is needed, which seems to be achievable by FFF, well-balancing the build rate with print resolution and, thus, qualifying it as promising technology to obtain shape memory polymers in entirely new shapes.

## 4. Conclusions

Fused filament fabrication is a suitable technique to produce bicolored additively manufactured QR code carriers in a dual extrusion process as demonstrated for a polyester urethane, which was used as model compound. The print resolution both in the XY-plane with regard to the QR code pattern and in Z-direction with reference to the layer height could be controlled by the experimental setup and the print instructions. This way, filigree, well-resolved structures could be obtained. The objects were able to resist strong deformations and characterized by distinct shape memory properties. Even in a multiple cycle experiment no major damage could be witnessed for the print objects. The use of congruence measurements has proven to be a valuable tool to determine the printing accuracy and shape recoverability. Although a higher resolution of the QR code pattern was achieved when using a setup with a 100 µm nozzle, with extending the production time, FFF seems to be a practical method in this scenario as well, which may give access to other technically demanding objects. The main advantages of the new manufacturing process for QR code carriers are that polymer extrusion can be easily controlled, a significantly lower amount of base material is needed, facilitating the fabrication of very thin layers with a thickness below 10 µm, and the use of solvents can be avoided. The latter is of ecological importance. All these aspects emphasize that the novel production process for QR code carriers is not only attractive for research purposes, but also from an economic point of view, not least because the material could be qualified for processing with a commercially available 3D printer. Therefore, FFF could turn out as an enabling technology to realize applications for SMPs in fields like counterfeit-proof marking of goods at risk of plagiarism and supervision of cold chains. Future challenges consist in shortening the production time without compromising on resolution and using the dimension of time to autonomously manipulate 3D printed objects, which is also known as 4D-printing, thus eliminating the need for programming.

## Figures and Tables

**Table 1:** Printing instructions for Ultimaker 3 to produce the three different prototypes of QR code carriers based on PEU.

Specifications	Substrate (Non-Dyed PEU)		Elevation (Red PEU)	
Type of QR code carrier	1, 2	3	1	2, 3
Diameter of the nozzle (µm)	400	400	100	400
Temperature of the nozzle (°C)	225	225	190	190
Speed of print head (mm·s^−1^)	50	50	4	7
Build rate (ml·h^−1^)	34.2	34.2	5.4	22.8
Build platform temperature (°C)	23	23	23	23
Number of layers	1	1	3	1
Layer height (µm)	180	15	63	190
